# Headache knowledge of young neurologists in Germany

**DOI:** 10.1186/s12909-026-09142-6

**Published:** 2026-04-11

**Authors:** Victoria Ruschil, Cem Thunstedt, Katharina Kamm

**Affiliations:** 1 Department of Neurology and Stroke, Hertie Center for Neurology, University Hospital Tuebingen, Tuebingen, Germany; 2 Center for rare diseases, University Hospital Tuebingen, Tuebingen, Germany; 3 Department of Neurology, LMU Klinikum, Ludwig-Maximilians-University Munich, Munich, Germany

**Keywords:** Headache, Migraine, Education, Medical training, Residency, Neurology

## Abstract

**Background:**

Primary and secondary headaches are a common complaint in neurology. Nonetheless, headache care remains suboptimal for various reasons. This study aimed to assess the training and expertise in headache medicine among young neurologists in Germany.

**Methods:**

From January 2023 to September 2024, we conducted a national, cross-sectional survey among neurology residents and young neurologists in Germany. Participants completed a questionnaire assessing self-reported training experience, knowledge of headache diagnosis and treatment and compliance to treatment guidelines. Data were analyzed using descriptive statistics and group comparisons based on training status.

**Results:**

A total of 93 participants (73 residents and 20 consultants, male = 44, 33.4 ± 4.8 years) were included. Most participants (89.2%) regularly treated headache patients, predominantly in emergency settings. While knowledge was highest for frequent primary headaches, deficits were evident for rare and secondary headache syndromes. Significant differences in knowledge were observed according to prior headache training. Although 67.4% of participants were aware of treatment guidelines, only 26.1% of participants have read them. 96.8% of participants considered headache training important, yet only 46.7% of participants felt well-trained. Lack of supervision (74.3%) and teaching (64.9%) were key barriers to education. Significant gaps in knowledge and adherence to guidelines were identified, particularly for medication overuse headache and cluster headache. Notably, headache medicine ranked lowest in perceived prestige among neurological subspecialties.

**Conclusions:**

Early-career German neurologists demonstrated sufficient knowledge of common primary headaches, however deficiencies remained for rare primary and secondary headache disorders. Improved, structured headache training during residency may improve guideline adherence and optimize patient care.

**Supplementary Information:**

The online version contains supplementary material available at 10.1186/s12909-026-09142-6.

## Background

 Headache is a prevalent symptom as main complaint or accompanying symptom in many other neurological conditions. Worldwide, up to 50% of adults experience headache at least once a year [1]. Headache is a frequent reason for consultation and 0.5–4% of all emergency visits are due to acute headache [2, 3]. Different studies investigated the frequency of primary and secondary headaches at emergency departments and showed that primary headaches are diagnosed in 39.5–59.4% and secondary headache occurs in 25.9–39.5% of headache patients presenting to emergency departments due to acute headache [2, 4].

The high prevalence of headache disorders and their frequent occurrence in other neurological conditions underline the importance of diagnostic and treatment knowledge.

However, there remains a notable shortcoming of diagnosis and treatment associated with frequent consultations due to ongoing disability and headache progression. It was found that 34.2% of migraine patients are not treated according to treatment guidelines and an European survey found that only 11% and 2.4% of German migraine patients received triptans and preventive medication although indicated [5, 6].

Migraine patients presenting to a tertiary outpatient headache center stated 7.2 ± 10.0 visits to different specialists one year prior to study inclusion and 31.3% of patients presented at least once to an emergency department, on average 4.1 ± 7.4 times [6].

One reason for these restrictions might be limitations in headache training as limited headache knowledge of residents has been shown before in different European countries [7–9].

The aim of this online, questionnaire-based study was to assess the current level of training and knowledge of headache disorders among neurology residents and young neurologists in Germany.

## Methods

### Residency in neurology

Residency is completed over a minimum of five years following medical school in Germany. The training is mostly conducted at a municipal or university hospital and can partially be completed at neurological private practices. During this training, mandatory rotations and various skills must be completed according to the regulations of the respective federal state. In general, residents must be trained in inpatient care of neurological patients, neurological intensive care, and diagnostic techniques like electroencephalography and electromyography / neurography. Advanced training in headache medicine is not required. The different training settings cause a heterogeneous level of expertise regarding different neurological disorders among residents.

### Study design

The study was approved by the Ethics Committee of the Ludwig Maximilian University of Munich (22-1081) and conducted in accordance with the Declaration of Helsinki. The study was designed as national, cross-sectional, online survey of residents in neurology and young neurologists in Germany. Subjects were informed and invited to participate in the study via e-mail to the directors of German neurology departments, who were asked to forward the invitation to their medical staff. Additionally, the survey link was published on the news page of the German Society of Neurology (https://dgn.org/artikel/aktuelle-umfrage-zu-diagnostik-und-behandlung-von-kopfschmerzen).

Participants were included in the analysis if they were residents in neurology or neurologists for less than three years. Questionnaires with missing data more than 50% of headache knowledge and treatment were excluded. Single missing items were allowed. The study was performed and reported according to the STROBE guidelines for observational studies.

### Questionnaire

The questionnaire was developed for this study by the authors (KK and VR) based on similar surveys conducted among residents in France, Denmark, and Norway [7–9]. An English version was uploaded as supplementary file (Supplements). The online survey was conducted using REDCap (Research Electronic Data Capture) [10, 11]. Participants were informed about the purpose of the study at the beginning of the questionnaire. After their consent, subjects participated anonymously. The questionnaire was divided in three sections. The first section collected demographic and professional data, including age, gender, training institution, level of training, and areas of interest. The second section focused on questions regarding the participants’ training opportunities in the field of headache medicine as well as a self-assessment of their knowledge of headache disorders. The third section comprised knowledge-based questions of the diagnosis and treatment of headache disorders.

### Statistical analysis

Data is presented as descriptive statistics (mean ± SD, numbers and percentages of participants). Group differences between personal headache knowledge were assessed using a Kruskal-Wallis-ANOVA followed by a Dunn-Bonferroni post hoc test if appropriate. For group differences of diagnostic work-up and treatment between headache training groups chi-square tests were used. Statistical analyses were performed using SPSS Statistics (IBM Corp. Released 2023. IBM SPSS Statistics for Windows, Version 29.0.2.0 Armonk, NY: IBM Corp). Significance was accepted at *p* < 0.05 (two-tailed).

## Results

### Participants

**Fig. 1:**
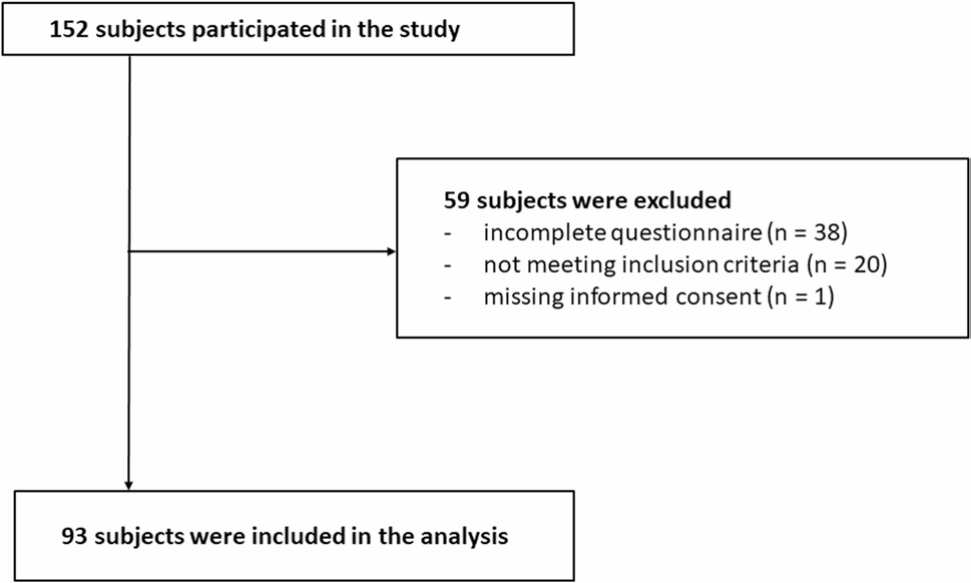
Participant disposition. 93 neurology residents or young consultants in neurology were included in the analysis. 59 participants were excluded

152 participants answered the questionnaire online between January 2023 and September 2024. Of these, 59 participants were excluded due to incomplete data (*n* = 38), non-fulfillment of inclusion criteria (*n* = 20) or missing informed consent (*n* = 1). 93 participants (male = 44, 33.4 ± 4.8 years) were included in the analysis (Fig. 1). 73 participants (male = 29, 32.5 ± 4.6 years) were residents in neurology and fulfilled 4.4 ± 2.4 years of residency. 20 participants (male = 15, 36.7 ± 4.1 years) were consultants in neurology for 1.6 ± 0.8 years (Table 1). 93.5% of participants worked at a hospital, 6.5% of participants worked in ambulatory care.

**Table 1:** Descriptive data of the study sample

	Headache specialist trained (n = 13)	Headache trained (n = 54)	Residency without headache training(n = 26)	
Sex (n, %)				
Male	7 (53.8%)	23 (42.6%)	14 (53.8%)	
Female	6 (46.2%)	30 (55.6%)	12 (46.2%)	
Divers	0 (0.0%)	1 (1.9%)	0 (0.0%)	
Age (years)	33.7 ± 4.1	32.8 ± 4.7	34.4 ± 5.9	p = 0.407
Resident (n, %)	10 (13.7%)	48 (65.8%)	15 (20.5%)	
Consultant (n, %)	3 (15.0%)	6 (30.0%)	11 (55.0%)	
Years of training (years)*	5.2 ± 3.1	4.4 ± 2.4	4.0 ± 2.2	p = 0.465
Years as consultant (years)	1.3 ± 0.6	1.4 ± 0.5	1.9 ± 1.0	p = 0.492
Type of care (n, %)				
University hospital	11 (11.8%)	10 (10.8%)	8 (8.6%)	
Municipal hospital	14 (15.1%)	38 (40.9%)	2 (2.2%)	
Private practice	4 (4.3%)	2 (2.2%)	0 (0.0%)	
Pain/ rehabilitation clinic	0 (0.0%)	1 (1.1%)	1 (1.1%)	
Other	1 (1.1%)	1 (1.1%)	0	

Participants who worked at a headache outpatient center were assigned to the headache specialist trained group (group 1). Participants who were trained in headache medicine were assigned to the headache trained group (group 2) and if no training was available participants were assigned to the residency without headache training group (group 3)

Data are shown as mean ± standard or number (percentage) of participants

* Only residents were included in the analysis

89.2% of participants medicated headache patients on a regular basis during their clinical routine. Headache patients were most often seen at the emergency department (80.6%), followed by hospitalized patients (50.5%), outpatient patients (35.5%) and consultation patients hospitalized at other departments (35.5%).

Based on their headache experience, participants were divided in three groups: 13 participants (m = 7, 33.7 ± 4.1 years) were trained at a specialized headache outpatient clinic (group 1, *headache specialist trained*), 54 participants (male = 23, 32.8 ± 4.7 years) stated to be trained in headache diagnosis and treatment (group 2, *headache trained*) and 26 participants (male = 14, 34.4 ± 5.9 years) stated no training is available at their work place (group 3, *residency without headache training*). There was no significant difference in work experience between the groups.

### Headache knowledge

**Fig. 2:**
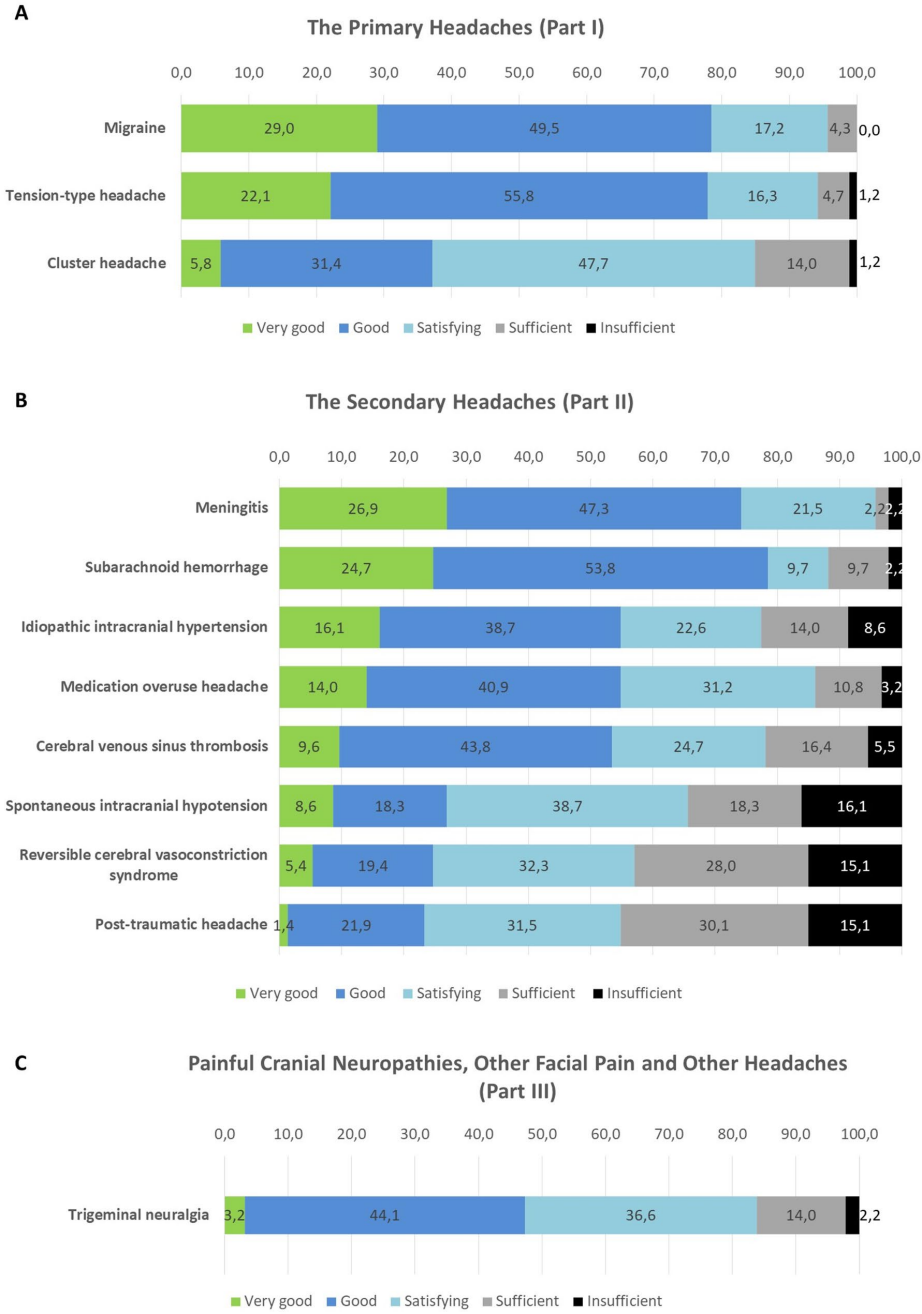
Rating of personal knowledge of headache disorders: A Primary headache disorders, B Secondary headache disorders, C Trigeminal neuralgia. Participants rated their personal knowledge of the above mentioned headache disorders from ‘very good’ to ‘insufficient’. The responses are given as percentage distribu-tions among the five rating categories (Green: very good, blue: good, light blue: satisfying, gray: sufficient, black: insufficient

Participants rated their knowledge highest for migraine and tension-type headache, lowest for cluster headache and trigeminal neuralgia. Concerning secondary headache participants stated highest knowledge in subarachnoid hemorrhage (SAH) and meningitis, lowest for reversible cerebral vasoconstriction syndrome (RCVS) and posttraumatic headache (Fig. 2).

Significant differences concerning knowledge between the above mentioned groups were shown for posttraumatic headache (Χ^2^ [2] = 10.955, *p* = 0.004) and RCVS (Χ^2^ [2] = 7.465, *p* = 0.024). Post-hoc tests showed significant differences between group 1 and 3 (z = 2.561, *p* = 0.031) and group 2 and 3 (z = 3.301, *p* = 0.003) for posttraumatic headache. Post-hoc tests showed significant differences between group 1 and 2 (z = 2.727, *p* = 0.019) for RCVS.

Questions concerning migraine prevalence and gender distribution were answered correctly by 67.0% and 48.4% of participants, respectively (Table 2). Cluster headache prevalence and gender distribution were answered correctly by 56.0% and 74.7% of participants, respectively, with no significant differences for migraine and cluster headache between the training groups. Prevalence of medication overuse (MOH) headache was answered correctly by 26.1% with a significant difference between the training groups (69,2% group 1, 20,4% group 2, 16% group 3; Χ^2^ [4] = 19.198, *p* < 0.001, *n* = 92).

**Table 2:** Headache knowledge and diagnostic work-up

	All (n=93), n (%)	Headache specialist trained(n=13), n (%)	Headache trained(n=54), n (%)	Residency without headache training (n=26), n (%)	
Migraine					
Prevalence					
0-5%	5 (5.5%)	1 (7.7%)	2 (3.8%)	2 (8.0%)	
5-10%	25 (27.5%)	3 (23.1%)	14 (26.4%)	8 (32.0%)	
10-15%*	61 (67.0%)	9 (69.2%)	37 (69.8%)	15 (60.0%)	*p* = 0.679
Gender ratio					
1:3*	44 (48.4%)	8 (61.5%)	28 (51.9%)	8 (33.3%)	
1:5	35 (38.5%)	4 (30.8%)	21 (38.9%)	10 (41.7%)	
1:10	12 (13.2%)	1 (7.7%)	5 (9.3%)	6 (25.0%)	*p *= 0.188
Cluster headache					
Prevalance					
1:10	3 (4.0%)	0 (0.0%)	2 (4.4%)	1 (5.9%)	
1:100	9 (12.0%)	0 (0.0%)	7 (15.6%)	2 (11.8%)	
1:1000*	42 (56.0%)	8 (66.7%)	26 (57.8%)	8 (47.1%)	
1:10000	20 (28.0%)	4 (33.3%)	10 (22.2%)	6 (35.3%)	*p* = 0.452
Gender ratio					
1:1	7 (9.3%)	1 (8.3%)	6 (13.3%)	0 (0.0%)	
4:1*	56 (74.7%)	10 (83.3%)	33 (73.3%)	13 (72.2%)	
10:1	12 (16.0%)	1 (8.3%)	6 (13.3%)	5 (27.8%)	*p* = 0.750
MOH					
Prevalence					
1%*	24 (26.1%)	9 (69.2%)	11 (20.4%)	4 (16.0%)	
5%	57 (62.0%)	3 (23.1%)	39 (72.2%)	15 (60.0%)	
10%	11 (12.0%)	1 (7.7%)	4 (7.4%)	6 (24.0%)	*p* < 0.001
Do you know the current guidelines for headache treatment of the German Society of Neurology (DGN) and the German Migraine and Headache Society (DMKG)?					
Yes, I know the guidelines	38 (41.3%)	4 (30.8%)	24 (44.4%)	10 (40.0%)	
Yes, I know the guidelines and have read them	24 (26.1%)	6 (46.2%)	14 (25.9%)	4 (16.0%)	
No	30 (32.6%)	3 (23.1%)	16 (29.6%)	11 (44.0%)	*p* = 0.290
Do you apply the current guidelines in clinical practice?					
Always	10 (16.1%)	30.8 (40.0%)	5 (13.2%)	1 (7.1%)	
More than half	36 (58.1%)	46.2 (60.0%)	22 (57.9%)	8 (57.1%)	
Less than half	10 (16.1%)	23.1 (30.0%)	6 (15.8%)	4 (28.6%)	
Rarely	6 (9.7%)	0 (0.0%)	5 (13.2%)	1 (7.1%)	
Never	0 (0.0%)	0 (0.0%)	0 (0.0%)	0 (0.0%)	*p* = 0.177
Do you use the International Classification of Headache Disorders (ICHD-III) regularly?					
Always	13 (14.1%)	5 (38.5%)	6 (11.1%)	2 (8.0%)	
More than half	23 (25.0%)	3 (23.1%)	15 (27.8%)	5 (20.0%)	
Less than half	14 (15.2%)	2 (15.4)	8 (14.8%)	4 (16.0%)	
Rarely	26 (28.3%)	1 (7.7%)	19 (35.2%)	6 (24.0%)	
Never	16 (17.4%)	2 (15.4%)	6 (11.1%)	8 (32.0%)	*p* = 0.075
Do you use headache diaries for diagnostic work-up on a regular basis?					
Always	13 (14.4%)	4 (30.8%)	6 (11.5%)	3 (12.0%)	
More than half	36 (40.0%)	6 (20.0%)	22 (42.3%)	8 (32.0%)	
Less than half	17 (18.9%)	1 (7.7%)	10 (19.2%)	6 (24.0%)	
Rarely	17 (18.9%)	2 (15.4%)	10 (19.2%)	5 (20.0%)	
Never	7 (7.8%)	0 (0.0%)	4 (7.7%)	3 (12.0%)	*p* = 0.614
Do you routinely record frequency of pain medication use?					
Always	51 (65.4%)	7 (53.8%)	32 (61.5%)	12 (63.2%)	
More than half	21 (26.9%)	0 (0.0%)	15 (28.8)	6 (31.6%)	
Less than half	5 (6.4%)	0 (0.0%)	4 (7.7%)	1 (5.3%)	
Rarely	0 (0.0%)	0 (0.0%)	0 (0.0%)	0 (0.0%)	
Never	1 (1.3%)	0 (0.0%)	1 (1.9)	0 (0.0%)	*p* = 0.590
Do you inform patients about the risks of medication overuse headache?					
Always	37 (40.7%)	6 (46.2%)	22 (41.5%)	9 (34.6%)	
More than half	39 (42.9%)	6 (46.2%)	23 (43.4%)	10 (40.0%)	
Less than half	10 (11.0%)	0 (0.0%)	6 (11.3%)	4 (16.0%)	
Rarely	5 (5.5%)	1 (7.7%)	2 (3.8%)	2 (8.0%)	
Never	0 (0.0%)	0 (0.0%)	0 (0.0%)	0 (0.0%)	*p* = 0.807
Do you regularly enquire about the impact of headaches on patients' quality of life?					
Always	18 (21.5%)	4 (36.4%)	9 (17.3%)	5 (23.8%)	
More than half	42 (50.0%)	4 (36.4%)	31 (59.6%)	7 (33.3%)	
Less than half	16 (19.0%)	2 (18.2%)	10 (19.2%)	4 (19.0%)	
Rarely	8 (9.5%)	1 (9.1%)	2 (3.8%)	5 (23.8%)	
Never	0 (0.0%)	0 (0.0%)	0 (0.0%)	0 (0.0%)	*p *= 0.108
Do you regularly ask patients to complete quality of life questionnaires (e.g. MIDAS, HIT-6, SF-12)?					
Always	1 (1.1%)	1 (7.7%)	0 (0.0%)	0 (0.0%)	
More than half	7 (7.7%)	5 (38.5%)	1 (1.9%)	2 (2.0%)	
Less than half	8 (11.0%)	2 (15.4%)	4 (7.5%)	4 (16.0%)	
Rarely	19 (20.9%)	3 (23.1%)	12 (22.6%)	4 (16.0%)	
Never	54 (59.3%)	2 (15.4%)	36 (67.9%)	16 (64.0%)	*p* < 0.001
Do you routinely record psychiatric comorbidities?					
Always	25 (27.5%)	6 (46.2%)	15 (28.3%)	4 (16.0%)	
More than half	40 (44.0%)	5 (38.5%)	20 (37.7%)	15 (60.0%)	
Less than half	16 (17.6%)	2 (15.4%)	10 (18.9%)	4 (16.0%)	
Rarely	10 (11.0%)	0 (0.0%)	8 (15.1%)	2 (8.0%)	
Never	0 (0.0%)	0 (0.0%)	0 (0.0%)	0 (0.0%)	*p* = 0.281
Do you regularly ask headache patients about the frequency of days absent from work?					
Always	7 (7.6%)	2 (15.4%)	2 (3.7%)	3 (12.0%)	
More than half	21 (22.8%)	4 (30.8%)	13 (24.1%	4 (16.0%)	
Less than half	22 (23.9%)	4 (30.8%)	11 (20.4%)	7 (28.0%)	
Rarely	32 (34.8%)	2 (15.4%)	23 (42.6%)	7 (28.0%)	
Never	10 (10.9%)	1 (7.7%)	5 (9.3%)	4 (16.0%)	*p* = 0.443

Participants were asked about epidemiology of headache disorders and headache treatment. The table shows the answers of all participants, further they are compared between the three training groups

* Correct answer

### Diagnostic work-up of headache patients

67.4% of participants stated to know current treatment guidelines in headache, 26.1% of par-ticipants read them before (Table 2). 46 participants (50.0%) used these guidelines, 39.1% of participants used International Classification of Headache Disorders 3rd edition (ICHD-3) crite-ria for diagnosis and 54.4% of participants used headache diaries on a regular basis (more than 50% in their daily routine).

92.3% of participants asked their patients about the frequency of acute medication intake and 83.6% of participants informed their patients about overuse of acute medication. There were no differences between the training groups.

Headache-related impact on quality of life was asked by 71.5% of participants. 8.8% of participants used patient-reported outcome measures on a regular basis, which differed significantly between the training groups (46,2% group 1, 1,9–2,0% group 2 and 3, Χ^2^ [8] = 31.513, *p* < 0.001, *n* = 92).

Psychiatric comorbidities were raised by 71.5% and absent days were asked by 30.4% of participants, with no difference between the training groups.

### Medical Treatment

#### Episodic migraine with low frequency

**Fig. 3:**
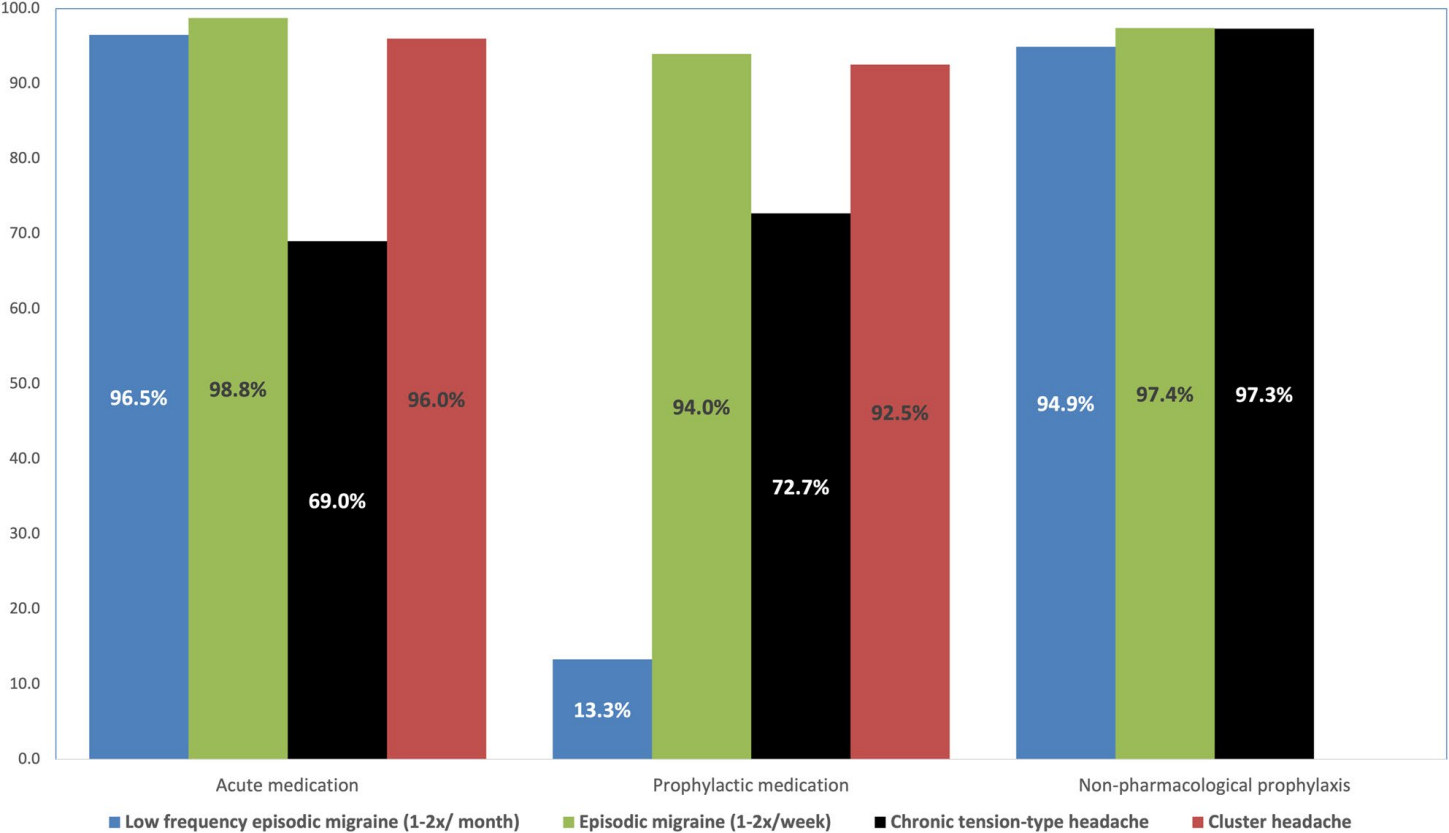
Treatment of primary headache disorders. Participants were asked about their pharmacological and non-pharmacological treatment choice for migraine, tension-type headache and cluster headache (Blue: low frequency episodic migraine (1-2x/ month); green: episodic migraine (1-2x/week); black: chronic tension-type headache, red: cluster headache)

Migraine attacks with low frequency (1–2 migraine attacks per month) were stated to be treated with acute medication by 96.5%, with prophylactic medication by 13.3% and with non-pharmacological treatment by 94.9% of participants, with no significant difference between the training groups (Fig. 3). Triptans (*n* = 56), ibuprofen (*n* = 51), aspirin (*n* = 47) and metamizole (*n* = 32) were chosen most often (Table 2). Aspirin (*p* = 0.049), metamizole (*p* = 0.032) and diclofenac (*p* = 0.002) were chosen significantly different between the training groups, most often by the *headache specialist trained* group. Preventive treatment was chosen by the *residency without headache training* and *headache trained* groups, commonly beta blocker (*n* = 10), followed by amitriptyline (*n* = 6), topiramate (*n* = 2) and valproate (*n* = 1). There was no significant difference between the two training groups concerning the choice of preventive treatment.

#### Episodic migraine

The majority of participants stated to treat episodic migraine patients with 1–2 migraine attacks per week using acute medication (98.8%), preventive medication (94.0%) and non-pharmacological treatment (97.4%) (Fig. 3). There were no significant differences between training groups.

Episodic migraine was treated most often by triptans (*n* = 70), ibuprofen (*n* = 44), aspirin (*n* = 41) and metamizole (*n* = 33) (Table 3). Significant differences between the training groups were shown for the use of diclofenac (*p* = 0.030) and naproxen (*p* = 0.018), more often chosen by the *headache specialist trained* group. For preventive treatment, most often beta blocker (*n* = 72), followed by amitriptyline (*n* = 28), topiramate (*n* = 24) and CGRP antibodies (*n* = 22) were chosen. Significant differences were shown for the use of flunarizine (*p* = 0.031) and amitriptyline (*p* = 0.028) that were chosen more often by the *headache specialist trained* group. Valproate (*n* = 7) and onabotulinumtoxin A (*n* = 5) were chosen as preventive treatment by few participants.

**Table 3:** Acute and preventive treatment for primary headache disorders

	All participants, n (%)	Headache specialist trained, n (%)	Headache trained, n (%)	Residency without headache training, n (%)	
Low frequency episodic migraine (1-2x/ month)					
Acute medication					
No	3 (3.5%)	0 (0.0%)	2 (3.8%)	1 (4.8%)	*p* = 0.744
Yes	83 (96.5%)	13 (100.0%)	50 (96.2%)	20 (95.2%)	
Prophylactic medication					
No	65 (86.7%)	13 (100.0%)	36 (81.8%)	16 (88.9%)	*p* = 0.226
Yes	10 (13.3%)	0 (0.0%)	8 (18.2%)	2 (11.1%)	
Non-pharmacological treatment					
No	4 (5.1%)	0 (0.0%)	2 (4.3%)	2 (10.0%)	*p* = 0.416
Yes	75 (94.9%)	13 (100.0%)	44 (95.7%)	18 (90.0%)	
Episodic migraine (1-2x/ week)					
Acute medication					
No	1 (1.2%)	0 (0.0%)	1 (2.0%)	0 (0.0%)	*p* = 0.718
Yes	80 (98.8%)	13 (100.0%)	48 (98.0%)	19 (100.0%)	
Prophylactic medication					
No	5 (6.0%)	0 (0.0%)	4 (8.0%)	1 (5.0%)	*p* = 0.545
Yes	78 (94.0%)	13 (100.0%)	46 (92.0%)	19 (95.0%)	
Non-pharmacological treatment					
No	2 (2.6%)	0 (0.0%)	1 (2.1%)	1 (5.9%)	*p* = 0.573
Yes	75 (97.4%)	13 (100.0%)	46 (97.9%)	16 (94.1%)	
Chronic tension-type headache					
Acute medication					
No	22 (31.0%)	3 (25.0%)	15 (35.7%)	4 (23.5%)	*p* = 0.582
Yes	49 (69.0%)	9 (75.0%)	27 (64.3%)	13 (76.5%)	
Prophylactic medication					
No	21 (27.3%)	2 (16.7%)	15 (32.6%)	4 (21.1%)	*p* = 0.425
Yes	56 (72.7%)	10 (83.3%)	31 (67.4%)	15 (78.9%)	
Non-pharmacological treatment					
No	2 (2.7%)	0 (0.0%)	44 (100.0%)	2 (10.0%)	*p *= 0.059
Yes	73 (97.3%)	11 (100.0%)	0 (0.0%)	18 (90.0%)	
Cluster headache					
Acute Medication					
No	3 (4.0%)	0 (0.0%)	1 (2.2%)	2 (10.5%)	*p* = 0.230
Yes	72 (96.0%)	11 (100.0%)	44 (97.8%)	17 (89.5%)	
Prophylactic medication					
No	5 (7.5%)	1 (8.3%)	3 (7.9%)	1 (5.9%)	*p* = 0.958
Yes	62 (92.5%)	11 (91.7%)	35 (92.1%)	16 (94.1%)	
Low frequency episodic migraine (1-2x/ month)					
Acute medication					
Triptan	56 (60.2%)	11 (84.6%)	29 (53.7%)	16 (61.5%)	*p* = 0.122
Ibuprofen	51 (54.8%)	8 (61.5%)	31 (57.4%)	12 (46.2%)	*p* = 0.557
Metamizole	32 (34.4%)	8 (61.5%)	19 (35.2%)	5 (19.2%)	*p* = 0.032
Naproxen	12 (12.9%)	3 (23.1%)	4 (7.4%)	5 (19.2%)	*p* = 0.167
Paracetamol/ Acetaminophen	26 (28.0%)	5 (38.5%)	16 (29.6%)	5 (19.2%)	*p* = 0.413
Aspirin	44 (47.0%)	7 (53.8%)	30 (55.6%)	7 (26.9%)	*p *= 0.049
Aspirin+Paracetamol+Caffeine	22 (23.7%)	4 (30.8%)	12 (22.2%)	6 (23.1%)	*p* = 0.806
Diclofenac	2 (2.2%)	2 (15.4%)	0 (0.0%)	0 (0.0%)	*p* = 0.002
Ergotamine	0 (0.0%)	0 (15.4%)	0 (0.0%)	0 (0.0%)	-
Prophylactic medication					
CGRP antibody	0 (0.0%)	0 (0.0%)	0 (0.0%)	0 (0.0%)	-
Amitriptyline	6 (6.5%)	0 (0.0%)	5 (9.3%)	1 (3.8%)	*p* = 0.388
Beta blocker	9 (9.7%)	0 (0.0%)	8 (14.8%)	1 (3.8%)	*p* = 0.133
Flunarizine	0 (0.0%)	0 (0.0%)	0 (0.0%)	0 (0.0%)	-
Onabotulinumtoxin A	0 (0.0%)	0 (0.0%)	0 (0.0%)	0 (0.0%)	-
Topiramate	2 (2.2%)	0 (0.0%)	2 (3.7%)	0 (0.0%)	*p* = 0.478
Valproate	1 (1.1%)	0 (0.0%)	1 (1.9%)	0 (0.0%)	*p* = 0.694
Episodic migraine (1-2x/ week)					
Acute medication					
Triptan	70 (75.3%)	13 (100.0%)	39 (72.2%)	18 (69.2%)	*p* = 0.080
Ibuprofen	44 (47.3%)	8 (61.5%)	25 (46.3%)	11 (42.3%)	*p *= 0.512
Metamizole	33 (35.5%)	6 (46.2%)	19 (35.2%)	8 (30.8)	*p* = 0.637
Naproxen	13 (14.0%)	4 (30.8%)	3 (5.6%)	6 (23.1%)	*p* = 0.018
Paracetamol/ Acetaminophen	23 (24.7%)	5 (38.5%)	14 (25.9%)	4 (15.4%)	*p* = 0.276
Aspirin	41 (44.1%)	5 (38.5%)	29 (53.7%)	7 (26.9%)	*p* = 0.071
Aspirin+Paracetamol+Caffeine	25 (26.9%)	4 (30.8%)	15 (27.8%)	6 (23.1%)	*p* = 0.855
Diclofenac	4 (4.3%)	2 (15.4%)	0 (0.0%)	2 (7.7%)	*p* = 0.030
Ergotamine	2 (2.2%)	1 (7.7%)	1 (1.9%)	0 (0.0%)	*p* = 0.288
CGRP antibody	22 (23.7%)	5 (38.5%)	11 (20.4%)	6 (23.1%)	*p *= 0.386
Amitriptyline	28 (30.1%)	8 (61.5%)	13 (24.1%)	7 (26.9%)	*p* = 0.028
Beta blocker	72 (77.4%)	13 (100.0%)	42 (77.8%)	17 (65.4%)	*p* = 0.051
Flunarizine	10 (10.8%)	4 (30.8%)	3 (5.6%)	3 (11.5%)	*p* = 0.031
Onabotulinumtoxin A	5 (5.4%)	2 (15.4%)	2 (3.7%)	1 (3.8%)	*p* = 0.226
Topiramate	24 (25.8%)	5 (38.5%)	13 (24.1%)	6 (23.1%)	*p* = 0.529
Valproate	7 (7.5%)	2 (15.5%)	3 (5.6%)	2 (7.7%)	*p* = 0.483
Chronic tension-type headache					
Acute medication					
Triptan	3 (3.2%)	1 (7.7%)	1 (1.9%)	1 (3.8%)	*p* = 0.552
Ibuprofen	32 (34.4%)	8 (61.5%)	15 (27.8%)	9 (34.6%)	*p* = 0.071
Metamizole	28 (30.1%)	7 (53.8%)	16 (29.6%)	5 (19.2%)	*p *= 0.084
Naproxen	7 (7.5%)	2 (15.4%)	4 (7.4%)	1 (3.8%)	*p *= 0.436
Paracetamol/ Acetaminophen	28 (30.1%)	6 (46.2%)	15 (27.8%)	7 (26.9%)	*p *= 0.395
Aspirin	14 (15.1%)	4 (30.8%)	10 (18.5%)	0 (0.0%)	*p* = 0.022
Aspirin+Paracetamol+Caffeine	11 (11.8%)	1 (7.7%)	7 (13.0%)	3 (11.5%)	*p* = 0.868
Diclofenac	1 (1.1%)	1 (7.7%)	0 (0.0%)	0 (0.0%)	*p* = 0.045
Ergotamine	0 (0.0%)	0 (0.0%)	0 (0.0%)	0 (0.0%)	-
Prophylactic medication					
CGRP antibody	3 (3.2%)	0 (0.0%)	2 (3.7%)	1 (3.8%)	*p* = 0.777
Amitriptyline	49 (52.7%)	10 (76.9%)	28 (51.9%)	11 (42.3%)	*p* = 0.122
Beta blocker	10 (10.8%)	1 (7.7%)	7 (13.0%)	2 (7.7%)	*p* = 0.721
Flunarizine	3 (3.2%)	0 (0.0%)	3 (5.6%)	0 (0.0%)	*p *= 0.326
Onabotulinumtoxin A	4 (4.3%)	0 (0.0%)	3 (5.6%)	1 (3.8%)	*p* = 0.669
Topiramate	5 (5.4%)	1 (7.7%)	3 (5.6%)	1 (3.8%)	*p* = 0.878
Valproate	0 (0.0%)	0 (0.0%)	0 (0.0%)	0 (0.0%)	-
Cluster headache					
Acute medication					
Triptan s.c.	30 (32.3%)	8 (61.5%)	14 (25.9%)	8 (30.8%)	*p* = 0.047
Triptan nasal	40 (43.0%)	7 (53.8%)	21 (38.9%)	12 (46.2%)	*p* = 0.576
Oxygen	63 (67.7%)	11 (84.6%)	40 (74.1%)	12 (46.2%)	*p* = 0.016
Triptan oral	10 (10.8%)	3 (23.1%)	5 (9.3%)	2 (7.7%)	*p* = 0.296
Ibuprofen	8 (8.6%)	0 (0.0%)	8 (14.8%)	0 (0.0%)	*p* = 0.042
Metamizole	4 (4.3%)	0 (0.0%)	4 (7.4%)	0 (0.0%)	*p* = 0.221
Paracetamol/ Acetaminophen	3 (3.2%)	0 (0.0%)	3 (5.6%)	0 (0.0%)	*p* = 0.326
Diclofenac	2 (2.2%)	0 (0.0%)	2 (3.7%)	0 (0.0%)	*p* = 0.478
Ergotamine	2 (2.2%)	0 (0.0%)	2 (3.7%)	0 (0.0%)	*p* = 0.478
Indometacin	10 (10.8%)	2 (15.4%)	5 (9.3%)	3 (11.5%)	*p* = 0.805
Lidocaine nasal	22 (23.7%)	2 (15.4%)	13 (24.1%)	7 (26.9%)	*p* = 0.722
Prophylactic medication					
Verapamil	47 (50.5%)	9 (69.2%)	25 (46.3%)	13 50.0%)	*p* = 0.331
Topiramate	8 (8.6%)	1 (7.7%)	5 (9.3%)	2 (7.7%)	*p* = 0.965
Lithium	7 (7.5%)	2 (15.4%)	4 (7.4%)	1 (3.8%)	*p* = 0.436
CGRP antibody	5 (5.4%)	2 (15.4%)	2 (3.7%)	1 (3.8%)	*p* = 0.226
Valproate	6 (6.5%)	1 (7.7%)	3 (5.6%)	2 (7.7%)	*p* = 0.918
Ergotamine	4 (4.3%)	0 (0.0%)	4 (7.4%)	0 (0.0%)	*p* = 0.221
Onabotulinumtoxin A	0 (0.0%)	0 (0.0%)	0 (0.0%)	0 (0.0%)	-
Frovatriptan	1 (1.1%)	0 (0.0%)	1 (1.9%)	0 (0.0%)	*p* = 0.694
Naratriptan	5 (5.4%)	2 (15.4%)	4 (7.4%)	1 (3.8%)	*p* = 0.523
Indometacin	3 (3.2%)	1 (7.7%)	1 (1.9%)	1 (3.8%)	*p* = 0.552
Beta blocker	7 (7.5%)	0 (0.0%)	6 (11.1%)	1 (3.8%)	*p* = 0.278

Specific treatment choices for migraine, tension-type headache and cluster headache shown for all participants and compared between the three training groups. Participants could select multiple options. Not all participants answered every item, percentages were calculated based on the number of respondents per item

#### Chronic tension-type headache

Chronic tension-type headache was suggested to be treated with acute medication by 69.0%, preventive medication by 72.7% and non-pharmacological treatment by 97.3% of participants (Fig. 3). There were no significant differences concerning these therapeutic approaches between the training groups. Ibuprofen (*n* = 32), followed by metamizole (*n* = 28) and paracetamol (*n* = 28) were selected for acute treatment (Table 3). For preventive treatment amitriptyline (*n* = 49) and beta blocker (*n* = 10) were most often chosen; there were no significant differences in the use of preventive treatment.

#### Cluster headache

96.0% of participants reported to prescribe acute medication and 92.5% of participants stated to start preventive treatment, with no significant differences between the training groups (Fig. 3). Oxygen (*n* = 63), nasal triptan (*n* = 40), subcutaneous triptan (*n* = 30) and nasal lidocaine (*n* = 22) were chosen most often (Table 3). Subcutaneous triptan was chosen most often by the *headache specialist trained* groupwith a significant difference between the training groups (*p* = 0.047). 27 participants (29.0%) stated to treat a cluster headache attack using oral NSAIDs or other oral analgesics (e.g. diclofenac, metamizole, indomethacin). Verapamil (*n* = 47), followed by topiramate (*n* = 8) and lithium (*n* = 7) was chosen for preventive treatment with no significant differences between the training groups.

#### Medication overuse headache

The majority of participants were aware that the intake of acute medication can cause medication overuse headache and stated the treatment of medication overuse to be challenging (89.4%). 38.8% of participants marked correctly all acute medication causing medication overuse headache (Table 4). For both questions there were no significant differences between the training groups.

**Table 4:** Medication overuse headache

Medication	% of participants
Ergotamine	77.8%
Combination analgetics	98.7%
NSAIDs	100%
Opioids	76.3%
Paracetamol	92.4%
Triptans	87.8%

Percentage of participants regarding the drug as causing medication overuse headache

### Headache training

96.8% of participants regarded training in headache as important or very important and 72.0% of participants stated to be trained in headache diagnosis and treatment. However, only 46.7% of participants feel very well or well trained. Participants in the *residency without headache training* group reported to feel well or very well trained least often (34.6%), participants in the *headache specialist trained* group most often (77%) with a significant difference between the groups (Χ^2^ [6] = 14.232, *p* < 0.027, *n* = 92). 75.3% of participants stated training in headache should have a higher significance during residency. 66.7% of participants were interested in primary headaches and 38.7% of participants were interested in secondary headaches. Multiple entries were possible.

Participants were asked about obstacles they recognized in headache training: most often lacking supervision (74.3%), insufficient teaching during medical school (64.9%) and missing contact to headache patients beyond emergency department (64.4%) were reported with no difference between the training groups. 88.7% of participants wished further education.

**Fig. 4:**
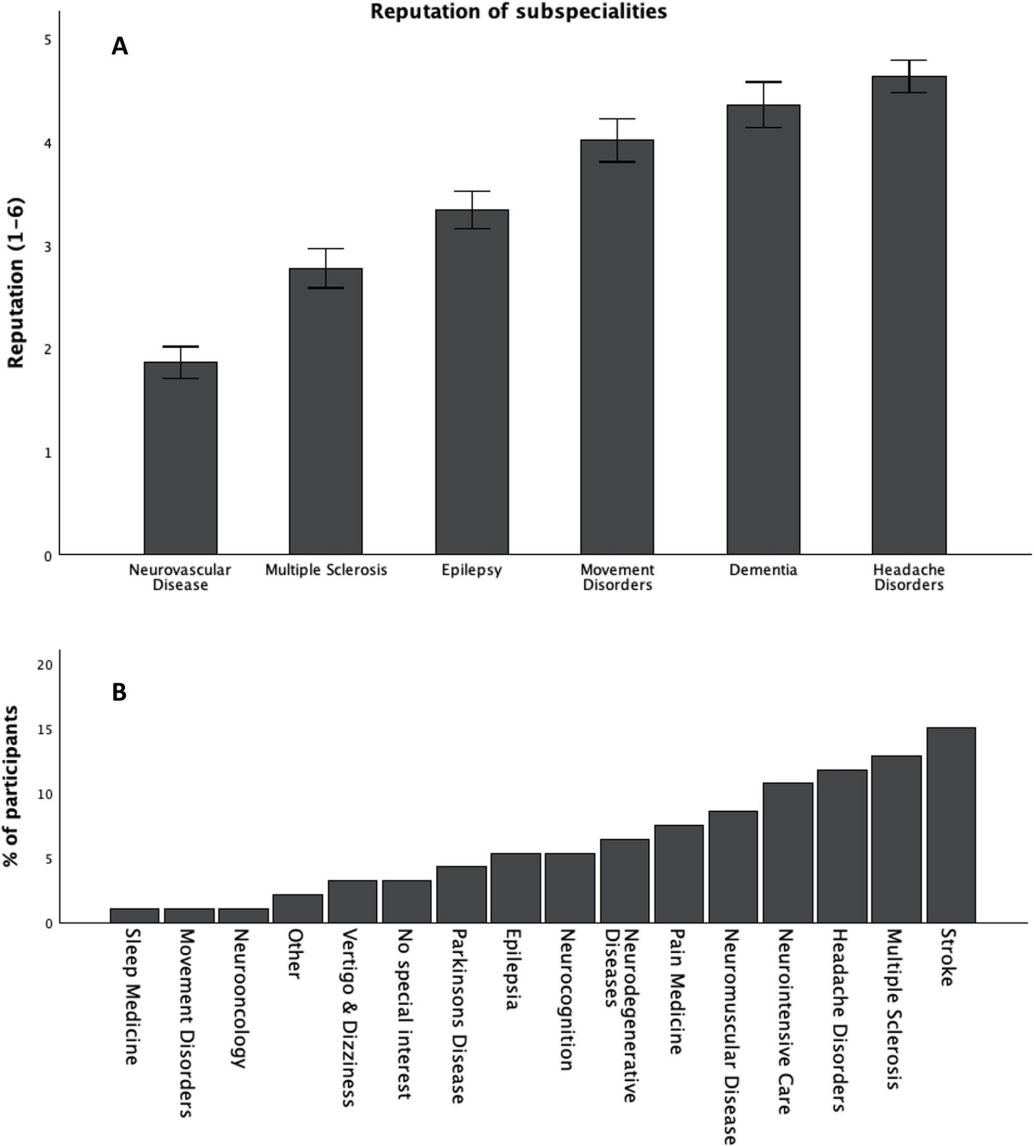
**A** Reputation of neurological disorders on a scale from 1 (‘highest reputation’) to 6 (‘lowest reputation’). Participants were asked to rate neurological subspecialties from ‘highest reputation (= 1)’ to ‘lowest reputation (= 6)’. **B** Interest in neurological subspecialty. Participants were asked to state the subspecialty with highest personal interest

On a scale from 1 (‘highest reputation’) to 6 (‘lowest reputation’), subjects rated the reputation of neurovascular disorders highest (1.8 ± 1.2) and headache lowest (4.3 ± 1.3) (Fig. 4A). Most participants were interested in vascular neurology (15.1%), multiple sclerosis (12.9%), headache (11.8%) and neurological intensive care (10.8%) (Fig. 4B).

## Discussion

The study reveals significant gaps in the training of headache disorders among residents and early-career neurologists in Germany. Whereas 96.8% of the participants regarded training in headache medicine as important, less than half of them felt well trained. At the same time, it could be shown that successful training has a positive impact on the competence in managing headache disorders as participants with specialized training felt significantly more competent than those without training.

Although participants self-reported to have good knowledge of frequent headache disorders like migraine or tension-type headache as well as for important secondary headaches like SAH or meningitis, for less common primary and secondary headache disorders, like cluster headache or RCVS, personal knowledge was rated low. Some knowledge-based questions also showed a discrepancy between self-assessment and the actual knowledge of certain aspects of frequent headache disorders. For example, only 50% of participants knew the correct gender distribution of migraine and the prevalence of MOH was answered correctly by less than 30% of respondents.

Concerning patient management, the study revealed less expertise in the collection of structured anamnestic data and use of diagnostic instruments than in pharmacological treatment of headache disorders. For example, only half of the participants stated to use headache diaries for diagnosis and documentation. Despite the recommended use of patient-reported outcome measures to assess the severity of headache and its progression under therapy, less than 10% stated to use them on a regular basis.

Moreover, the study indicates that guideline recommendations are taken into account by only 50% of the participants on a regular basis during their clinical routine while only 26.1% of participants have read the guidelines before. Further, ICHD-3-criteria are used by only 40% of participants for diagnostic purposes. The low adherence to guidelines or support material was not further investigated in this study, so possible reasons like time constraints or low adherence to clinical practice guidelines in general can only be speculated. It is known that adherence to clinical practice guidelines might be limited due to several reasons [12, 13], so the further implementation of the guidelines or spreading of possible benefits in patient care might be an approach to optimize headache care as well as reduce inappropriate use of health care resources.

In this study, episodic migraine was most often treated using triptans and NSAIDs. The high use of triptans as gold-standard treatment of acute migraine is an encouraging result that younger neurologists are aware of efficacious migraine therapy and use these early in treatment, however this might be also biased by the high interest in headache of participants.

Further, episodic migraine with 1–2 attacks/ week was treated with preventive medication by 94.0% of participants. Interestingly, the majority of participants used a traditional preventive medication, less often CGRP antibodies. This might reflect prescription requirements in Germany at the time of the survey or a higher familiarity with oral migraine preventive medication. Five participants chose onabotulinumtoxin A which is not indicated for episodic migraine maybe reflecting the low adherence to guideline recommendations.

Medication overuse headache was perceived as challenging by most participants, independent of their training. Encouragingly, about 90% of participants asked and informed their patients about the frequency and risks of acute medication intake. This finding is supported by the fact that most participants were aware of medication overuse headache caused by acute medication. Lower awareness for ergotamine and opioids might be due to the fact, that ergotamines and opioids are not commonly prescribed for headache patients in Germany.

For cluster headache, only 32.3% of participants recommended gold-standard sumatriptan and it was recommended significantly more often by the *headache specialist trained* group. Further, 27 participants suggested oral acute medication that cannot be recommended due to its long duration of action predominantly by the *residency without headache training* or *headache trained* group. These results indicates that headache training particularly affects the level of knowledge, especially of rare headache disorders.

Although headache is a prevalent complaint and headache disorders are common, participants rank this specialty with lowest prestige. Interestingly, these results are in line with former European surveys which also found headache and dementia the least prestigious subspecialities in neurology [7, 14]. The low prestige of headache medicine could also have an impact on education in this field and reasons for this evaluation should be further determined.

As a more structural reason for insufficient headache training, a lack of supervision was reported by almost 75% of participants as being the most critical factor contributing to poor training. Two-thirds of respondents also confirmed a lack of continuing education alongside their residency and not having enough insight into headache medicine apart from emergency situations.

### Strengths and limitations

To the best of our knowledge this study showed for the first time knowledge and reputation of headache medicine among German residents and young consultants in neurology. One strength of this study was the wide range of questions that have been raised, combining personal evaluation as well as theoretical and practical management of headache disorders.

One limitation might be the sample size, therefore some differences between the training groups didn’t reach significance. Further, there might be some selection bias due to an overrepresentation of physicians with a genuine interest in headache medicine being more motivated to participate in the study than those primarily interested in other fields of neurology.

Another limitation might be the low representation of the ambulatory sector, since the evaluation and treatment of headache patients is mainly a field of ambulatory medicine. Specialist training is mainly provided by hospitals in Germany, so the limited contact with routine care of headache patients could be a barrier to the education of young neurologists in the field of headache medicine. This topic should be addressed in future studies.

## Conclusions

The study highlights the need for an enhanced training of headache medicine in neurology in Germany. Even though there is basic knowledge on common headache disorders, insufficient education especially reflects a low expertise in less common headache disorders and the management of headache patients under consideration of clinical guidelines.

## Supplementary Information

Supplementary Material 1.

## Data Availability

The datasets used and analyzed during the current study are available from the corresponding author on reasonable request.

## Abbreviations

SAH: Subarachnoid Hemorrhage
RCVS: Reversible Cerebral Vasoconstriction Syndrome
MOH: Medication Overuse Headache
ICHD-3: International Classification of Headache Disorders, 3rd edition
REDCap: Research Electronic Data Capture
SD: Standard Deviation
NSAIDs: Non-Steroidal Anti-Inflammatory Drugs
CGRP: Calcitonin Gene-Related Peptide
