# Supplementary material for: Headache knowledge of young neurologists in Germany
Source: BMC Med Educ. 2026 Apr 11;26:636. doi: 10.1186/s12909-026-09142-6 (PMC13091273; doi:10.1186/s12909-026-09142-6)
Supplement: Supplementary file 1 — Supplementary Material 1. [file 12909_2026_9142_MOESM1_ESM.docx]

**Headache training in neurological residency in Germany - Questionnaire**

Thank you for your interest in actively participating in our research. Please read the following information carefully and confirm your participation in our study if you wish to do so. Participation is voluntary. You will only be included if you give your consent. If you have any further questions about the study, please do not hesitate to contact us.

**Aims of the study:**

The prevalence of headache is high in Germany, with approximately 60% of people over the age of 18 years suffering from headache at least once a year. Headache is one of the most common reasons for visiting emergency services and general practitioners. In this respect, headache is a key clinical picture for neurologists. This study aims to examine the level of training in relation to the symptom of headache in specialist neurological training.

**Who can participate?**

-Resident physicians in neurology

-Specialists in neurology

**Study procedure:**

This study is a questionnaire study. At the beginning, we ask you to give your consent (by ticking the box provided) to participate in the study. The questionnaire will only be evaluated if a declaration of consent has been provided. The survey, evaluation and publication is ANONYMOUS.

If you decide to participate, we ask you to carefully complete the following questionnaire; this will take approximately 20 minutes.

**I. Personal details**

**1. Please indicate your age in years.**

**2. Please indicate your gender.**

- Male
- Female
- Diverse

**3. In which federal state of Germany do you work?**

- Baden-Württemberg
- Bavaria
- Berlin
- Brandenburg
- Bremen
- Hamburg
- Hesse
- Mecklenburg-Western Pomerania
- Lower Saxony
- North Rhine-Westphalia
- Rhineland-Palatinate
- Saarland
- Saxony-Anhalt
- Saxony
- Schleswig-Holstein
- Thuringia

**II. Training & Current Position**

**4. Please select your current level of education:**

- Resident
- Specialist

**4.1 For Residents: Which year of your continuing education (full years since starting your career) are you currently in?**

- < 6 months
- 6 - 12 months
- 1 year
- 2 years
- 3 years
- 4 years
- 5 years
- > 5 years

**4.2 For Residents: Please indicate, if applicable, the duration of any periods without continuing education (e.g., parental leave, research activities) during your specialist training:**

- < 6 months
- 6 - 12 months
- 1 year
- 2 years
- 3 years
- 4 years
- 5 years
- > 5 years

**4.3. For Residents: Please indicate approximately how long it will take you to become a specialist from now on:**

- < 6 months
- 6 - 12 months
- 1 year
- 2 years
- 3 years
- 4 years
- 5 years
- > 5 years

**4.4. For specialists: Please indicate how many years you have been a specialist in neurology (full years since qualifying as a specialist):**

- 1
- 2
- 3
- 4
- 5
- 6
- 7
- 8
- 9
- 10
- >10

**5. Please indicate where you’re currently employed:**

- Private practice
- Medical care center
- Pain clinic/rehabilitation clinic
- Primary and standard care facility
- University hospital
- Maximum care hospital
- Research
- Currently not working as a physician
- Maternity leave/parental leave
- Other occupation

**6. Did you work at another hospital during your training as a specialist?**

- Yes
- No

**7. What is your career goal?**

- Specialist physician working in a hospital
- Senior physician working in a hospital
- Chief physician working in a hospital
- Employed specialist physician working in a practice
- Self-employed specialist physician in a practice
- Academic career with habilitation/professorship
- Alternative career goals (e.g., pharmaceutical industry, consulting)
- Other
- Undecided

**8. Please indicate ONE specialization in neurology that interests you most.**

- Movement disorders
- Epilepsy
- Neurocognition
- Headache
- Parkinson's disease
- Multiple sclerosis
- Neurodegeneration
- Neurointensive care
- Neuromuscular diseases
- Neurooncology
- Neuropathology
- Neurorehabilitation
- Sleep medicine
- Stroke
- Pain medicine
- Vertigo & Dizziness
- Other
- No special interest

**9. Please list ALL specializations in neurology that interest you.**

- Movement disorders
- Epilepsy
- Neurocognition
- Headache
- Parkinson's disease
- Multiple sclerosis
- Neurodegeneration
- Neurointensive care
- Neuromuscular diseases
- Neurooncology
- Neuropathology
- Neurorehabilitation
- Sleep medicine
- Stroke
- Pain medicine
- Vertigo & Dizziness
- Other
- No special interest

**10. Please indicate which of the following specializations you believe enjoy the highest reputation in neurology. Please rank them from 1 (highest reputation) to 6 (lowest reputation).**

- Neurovascular disease
- Dementia
- Epilepsy
- Headache
- Multiple sclerosis
- Movement disorders

**III. Headache Training & Treatment of Headache Patients**

**11. How important do you consider headache training to be for your professional work?**

- Very important
- Important
- Neither important nor unimportant
- Somewhat unimportant
- Unimportant

**12. Are you trained at your workplace in the diagnosis and treatment of headache disorders?**

- Yes
- No

**13. Is there a special consultation for headache patients at your workplace?**

- Yes
- No

**13.1 If yes, do you have the opportunity to rotate there as part of your residency?**

- Yes
- No

**14. Have you already worked in this special consultation hour?**

- Yes
- No

**14.1 If yes, please indicate how long you have worked there:**

- 1 - 3 months
- 4 - 6 months
- 6 - 9 months
- 9 - 12 months
- 1 - 1.5 years
- 1.5 - 2 years
- 2 - 2.5 years
- 2.5 - 3 years
- > 3 years

**15. How well trained do you feel in terms of diagnosing and treating headache disorders?**

- Very well
- Well
- Moderately
- Poorly
- Very poorly

**16. Do you regularly see headache patients in your daily clinical routine?**

- Yes
- No

**17. Do you think headache disorders should be given greater priority in specialist training for neurologists?**

- Yes
- No

**17.1 If yes: Where do you encounter headache patients in your daily clinical practice?**

- During outpatient consultations
- During consultations in hospital
- On the ward
- In emergency care
- In consultation services

17.1.1. For consultation (outpatient, hospital, emergency): How often do you see headache patients during consultation?

- Daily
- Regularly
- Frequently
- Rarely
- Never

**18. What diagnosis do you make most often?**

- Tension-type headache
- Migraine
- Cluster headache
- Other diagnosis

**18.1 If other diagnosis: Please indicate which diagnosis you make most frequently? (free text)**

**19. How would you rate your own knowledge concerning the below listed headache disorders?**

| **Headache** | **Very good** | **Good** | **Satisfying** | **Sufficient** | **Insufficient** |
| --- | --- | --- | --- | --- | --- |
| Migraine | **o** | **o** | **o** | **o** | **o** |
| Tension-type headache | **o** | **o** | **o** | **o** | **o** |
| Cluster headache | **o** | **o** | **o** | **o** | **o** |
| Medication overuse headache | **o** | **o** | **o** | **o** | **o** |
| Trigeminal neuralgia | **o** | **o** | **o** | **o** | **o** |
| Post-traumatic headache | **o** | **o** | **o** | **o** | **o** |
| Cerebral venous sinus thrombosis | **o** | **o** | **o** | **o** | **o** |
| Subarachnoid hemorrhage | **o** | **o** | **o** | **o** | **o** |
| Reversible cerebral vasoconstriction syndrome | **o** | **o** | **o** | **o** | **o** |
| Meningitis | **o** | **o** | **o** | **o** | **o** |
| Idiopathic intracranial hypertension | **o** | **o** | **o** | **o** | **o** |
| Spontaneous intracranial hypotension | **o** | **o** | **o** | **o** | **o** |

**20. Do you use the International Classification of Headache Disorders, 3^rd^ edition (ICHD-III), for diagnosis of headache disorders?**

- Always
- More than half the time
- Less than half the time
- Rarely
- Never

**21. Do you know the current guidelines for headache treatment of the German Society of Neurology (DGN) and the German Migraine and Headache Society (DMKG)?**

- Yes, I know the guidelines
- Yes, I know the guidelines and have read them
- No

**21.1 If yes, do you apply the current guidelines in clinical practice?**

- - Always
  - More than half the time
  - Less than half the time
  - Rarely
  - Never

**22. How do you estimate the prevalence of migraine in Germany?**

- 0 - 5%
- 5 - 10%
- 10 - 15%

**23. In your opinion, what is the ratio of men to women among adult migraine patients in Germany?**

- 1/3
- 1/5
- 1/10

**24. Do you use headache diaries for diagnostic work-up on a regular basis?**

- Always
- More than half the time
- Less than half the time
- Rarely
- Never

**25. Do you routinely record frequency of pain medication use?**

- Always
- More than half the time
- Less than half the time
- Rarely
- Never

**26. How do you estimate the prevalence of medication overuse headache in Germany?**

- ca. 1%
- ca. 5 %
- ca. 10%

**27. Do you routinely record psychiatric comorbidities?**

- Always
- More than half the time
- Less than half the time
- Rarely
- Never

**28. In your opinion, what percentage of migraine patients also suffer from depression?**

- 0 - 20%
- 20 - 40%
- 40 - 60%
- > 60%

**29. Do you inform patients about the risks of medication overuse headache?**

- Always
- More than half the time
- Less than half the time
- Rarely
- Never

**30. Do you regularly enquire about the impact of headache on patients' quality of life?**

- Always
- More than half the time
- Less than half the time
- Rarely
- Never

**31. Do you regularly ask patients to complete quality of life questionnaires (e.g. MIDAS, HIT-6, SF-12)?**

- Always
- More than half the time
- Less than half the time
- Rarely
- Never

**32. Do you regularly ask headache patients about the frequency of days absent from work?**

- Always
- More than half the time
- Less than half the time
- Rarely
- Never

**How do you usually treat migraine patients with 1-2 migraine attacks per week?**

**33. Acute medication**

- Yes
- No

**33.1. If yes,**

- - Aspirin
  - Diclofenac
  - Triptan
  - Ibuprofen
  - Metamizole/Novaminsulfon
  - Naproxen
  - Paracetamol/ Acetaminophen
  - Aspirin + Paracetamol + Caffeine
  - Ergotamine

**34. Prophylactic medication**

- Yes
- No

**34.2. If yes,**

- - Beta blocker (e.g., metoprolol, propranolol)
  - Flunarizine
  - Valproate
  - Topiramate
  - Amitriptyline
  - Onabotulinumtoxin A
  - CGRP antibody
  - Other medication(s)

**35. Non-pharmacological treatment**

- Yes
- No

**How do you usually treat migraine patients with a migraine attack every 1-2 months?**

**36. Acute medication**

- Yes
- No

**36.1 If yes,**

- - Aspirin
  - Diclofenac
  - Triptan
  - Ibuprofen
  - Metamizole/Novaminsulfon
  - Naproxen
  - Paracetamol/ Acetaminophen
  - Aspirin + Paracetamol + Caffeine
  - Ergotamine

**37. Prophylactic medication**

- Yes
- No

**37.1. If yes,**

- - Beta blocker (e.g., metoprolol, propranolol)
  - Flunarizine
  - Valproate
  - Topiramate
  - Amitriptyline
  - Onabotulinumtoxin A
  - CGRP antibody
  - Other medication(s)

**38. Non-pharmacological treatment**

- Yes
- No

**How do you usually treat a patient with chronic tension-type headache (> 15 headache days/month)?**

**39. Acute medication**

- Yes
- No

**39.1 If yes,**

- Aspirin
- Diclofenac
- Triptan
- Ibuprofen
- Metamizole/Novaminsulfon
- Naproxen
- Paracetamol/ Acetaminophen
- Combination analgesics: Aspirin+Paracetamol+Caffeine
- Ergotamine

**40. Prophylactic medication**

- Yes
- No

**40.1. If yes,**

- Beta blocker (e.g., metoprolol, propranolol)
- Flunarizine
- Valproate
- Topiramate
- Amitriptyline
- Onabotulinumtoxin A
- CGRP antibody
- Other medication(s)

**41. Non-pharmacological treatment**

- Yes
- No

**42. Do you consider patients with medication overuse headache to be challenging?**

- Yes
- No

**43. Do you think, it is possible that medication overuse headache could be caused by overusing the following medications?**

- Caffeine
- Ergotamine
- Combination analgesics (e.g., ASA + paracetamol + caffeine, etc.)
- NSAIDs
- Opioids
- Paracetamol
- Triptans
- Antidepressants (e.g., amitriptyline, duloxetine, or venlafaxine)
- Antihypertensives (e.g., beta blockers, angiotensin II receptor blockers, calcium channel blockers, etc.)
- Anticonvulsants (e.g., topiramate)
- Botulinum toxin

**44. Do you usually recommend one or more of the following measures for the treatment of medication overuse headache?**

- Steroids
- Treatment with acute medication
- Inpatient treatment
- Sick leave
- Other

**How do you normally treat a patient with cluster headache and 3 attacks per day over 2 months?**

**45. Acute Medication**

- Yes
- No

**45.1. If yes,**

- - Inhalation of oxygen
  - Diclofenac
  - Ergotamine
  - NSAIDs
  - Metamizole/Novaminsulfon
  - Lidocaine (nasal)
  - Paracetamol
  - Triptan (nasal spray)
  - Triptan (subcutaneous)
  - Triptan (tablet)
  - Combination analgesics (e.g., ASA + paracetamol + caffeine)
  - Indomethacin
  - Other medication(s)

**46. Prophylactic medication**

- Yes
- No

**46.1 If yes,**

- - Beta blockers (e.g., metoprolol, propranolol)
  - Verapamil
  - Indomethacin
  - Topiramate
  - Valproate
  - Ergotamine
  - Onabotulinumtoxin A
  - CGRP antibody
  - Frovatriptan
  - Naratriptan
  - Lithium
  - Other medication(s)

**47. How would you estimate the prevalence of cluster headache in the German population?**

- 1/10
- 1/100
- 1/1000
- 1/10000

**48. In your estimation, how does the proportion of men affected by cluster headache compare to women?**

- 1/1
- 4/1
- 10/1

**49. What do you consider to be the biggest obstacles to improve the treatment of your headache patients? Please rate the following answers as important, not important, or neither.**

|  | **Important** | **Not important** | **Neither** |
| --- | --- | --- | --- |
| Inadequate training during medical school | **o** | **o** | **o** |
| Lack of contact with headache patients during further training (outside of emergency care) | **o** | **o** | **o** |
| Inadequate supervision | **o** | **o** | **o** |
| Lack of effective treatment options for the majority of patients | **o** | **o** | **o** |
| Lack of opportunities for follow-up appointments in outpatient/private practice settings | **o** | **o** | **o** |

**50. Would you like further training and continuing education opportunities beyond those currently available to you?**

- Yes
- No

**50.1. If yes, in terms of content, I am interested in...**

- - Diagnosis and treatment of primary headaches, such as migraine or cluster headache
  - Diagnosis and treatment of secondary headaches, such as subarachnoid hemorrhage

**50.2 If yes, I would like to see…**

- More training opportunities offered by professional associations.
- The opportunity to observe at a headache center.
- A fixed rotation with a focus on headache care.
- free text

**51. If you have any further comments, please feel free to add them here (free text)**
